# The Use of Body Worn Sensors for Detecting the Vibrations Acting on the Lower Back in Alpine Ski Racing

**DOI:** 10.3389/fphys.2017.00522

**Published:** 2017-07-20

**Authors:** Jörg Spörri, Josef Kröll, Benedikt Fasel, Kamiar Aminian, Erich Müller

**Affiliations:** ^1^Department of Sport Science and Kinesiology, University of Salzburg Hallein-Rif, Austria; ^2^Department of Orthopedics, Balgrist University Hospital, Zurich, University of Zurich Zurich, Switzerland; ^3^Laboratory of Movement Analysis and Measurement, École Polytechnique Fédérale de Lausanne Lausanne, Switzerland

**Keywords:** injury prevention, overuse injuries, wearable sensors, spine, back pain, athletes, alpine skiing, training load management

## Abstract

This study explored the use of body worn sensors to evaluate the vibrations that act on the human body in alpine ski racing from a general and a back overuse injury prevention perspective. In the course of a biomechanical field experiment, six male European Cup-level athletes each performed two runs on a typical giant slalom (GS) and slalom (SL) course, resulting in a total of 192 analyzed turns. Three-dimensional accelerations were measured by six inertial measurement units placed on the right and left shanks, right and left thighs, sacrum, and sternum. Based on these data, power spectral density (PSD; i.e., the signal's power distribution over frequency) was determined for all segments analyzed. Additionally, as a measure expressing the severity of vibration exposure, root-mean-square (RMS) acceleration acting on the lower back was calculated based on the inertial acceleration along the sacrum's longitudinal axis. In both GS and SL skiing, the PSD values of the vibrations acting at the shank were found to be largest for frequencies below 30 Hz. While being transmitted through the body, these vibrations were successively attenuated by the knee and hip joint. At the lower back (i.e., sacrum sensor), PSD values were especially pronounced for frequencies between 4 and 10 Hz, whereas a corresponding comparison between GS and SL revealed higher PSD values and larger RMS values for GS. Because vibrations in this particular range (i.e., 4 to 10 Hz) include the spine's resonant frequency and are known to increase the risk of structural deteriorations/abnormalities of the spine, they may be considered potential components of mechanisms leading to overuse injuries of the back in alpine ski racing. Accordingly, any measure to control and/or reduce such skiing-related vibrations to a minimum should be recognized and applied. In this connection, wearable sensor technologies might help to better monitor and manage the overall back overuse-relevant vibration exposure of athletes in regular training and or competition settings in the near future.

## Introduction

On the topic of the relationship between training load and sports injuries, there is emerging evidence that poor load management (i.e., an insufficient balance between loading and recovery) is a major injury risk factor (Drew and Finch, 2016). Accordingly, monitoring the external loads that act on the human body is key to better understanding the occurrence of (and potentially to avoid) injuries in competitive sports (Soligard et al., 2016). In this context, body worn inertial measurement units (IMU) may offer a pervasive way to measure both load-related body postures, as well as vibrations acting on the human body during outdoor sports activities (Kim et al., 1993; Chardonnens et al., 2013; Seel et al., 2014; Fasel et al., 2017). Moreover, they may provide important information regarding training or competition time, movement repetitions and/or the accelerations acting on the different segments of the human body (Chardonnens et al., 2012, 2014; Rawashdeh et al., 2016; Yu et al., 2016; Whiteside et al., 2017). Thus, particularly for investigating the link between load and injury, as well as for monitoring and/or managing training and competition load, sensor-based wearable technologies might serve as an essential tool in the near future. In the current study, their practical usefulness will be demonstrated through the sport of alpine ski racing.

In alpine ski racing, the relatively high risk of injury is well documented and recognized (Pujol et al., 2007; Flørenes et al., 2009; Westin et al., 2012; Bere et al., 2013). In recent years, substantial research efforts concerning injury causes and prevention measures have been undertaken (Spörri et al., 2016b). However, most alpine ski racing-related research has focused on traumatic injuries, while overuse injuries have received little attention (Supej et al., 2017). Accordingly, exploring the potential causes of overuse injuries in order to provide evidence-based recommendations for their prevention has been suggested to be an important task for the future alpine ski racing-related research agenda (Supej et al., 2017).

Similar to other competitive sports, in alpine ski racing the athlete's back has been reported to be one of those body parts that is particularly prone to overuse injuries (Bergstrom et al., 2004; Hildebrandt and Raschner, 2013; Spörri et al., 2015a). As early as adolescence, competitive alpine skiers were discovered to have significantly more prevalent radiographic abnormalities as non-athletic age-matched controls (Rachbauer et al., 2001; Todd et al., 2015). Furthermore, several studies have documented such abnormalities as being associated with a higher risk of developing low-back pain later, either during or after the sports career (Luoma et al., 2000; Lundin et al., 2001; Ogon et al., 2001; Iwamoto et al., 2004). From a biomechanical perspective, several factors may contribute to the development of overuse injuries of the back in alpine ski racing.

First, similar to other competitive sports, an accumulation of heavy mechanical loads exceeding the athletes' capacities, particularly if the recovery time between the loadings is insufficient, may lead to tissue damage and overuse injuries (Soligard et al., 2016). This appears quite plausible, as an association between cumulative low back loads and low back pain has already been demonstrated for different athletic (i.e., other than alpine skiing) and occupational cohorts (Kujala et al., 1996; Heneweer et al., 2011; Coenen et al., 2013).

Second, with the use of body worn sensors, recent studies of alpine ski racing explored that typical loading patterns of the back include a combined occurrence of frontal bending, lateral bending and torsion in the trunk, as well as high peak loads (Spörri et al., 2015a,b, 2016a). Since a combination of these factors is known to be related to high spinal disc loading (Nachemson, 1981; Wilke et al., 1999; Haid and Fischler, 2013), and has been suggested to be attributable to different types of spine deteriorations (Rachbauer et al., 2001; Hangai et al., 2009), they may be considered important mechanisms leading to overuse injuries of the back in alpine ski racing (Spörri et al., 2015a,b, 2016a).

Third, there is strong scientific evidence that excessive exposure to whole-body vibrations, particularly at frequencies close to the resonant frequency of the spine [~4–10 Hz according to Izambert et al. (2003), Guo et al. (2009), Guo et al. (2011), and Baig et al. (2014)], increases the risk of structural deteriorations/abnormalities of the spine and of developing low back pain (Hill et al., 2009; Burström et al., 2015). For that and other reasons, there are international standards such as, *ISO 2631* (ISO, 1997) or *Directive 2002/44/EC* of the European Union (EU, 2002) that define minimum health and safety requirements for the exposure of workers arising from whole-body vibrations (Griffin, 2004).

Regarding the vibrations that occur while skiing, earlier studies primarily focused on recreational skiing (Kugovnik et al., 2000; Federolf et al., 2009; Supej, 2013; Tarabini et al., 2015) and/or the ski-plate-binding-boot unit level (Kugovnik et al., 2000; Federolf et al., 2009; Tarabini et al., 2015). However, it is reasonable that vibrations in alpine ski racing are markedly different than those occurring in recreational skiing. Based on the preliminary findings of two pilot studies, it is known that vibrations are damped when being transmitted through the skier's body (Supej, 2013; Fasel et al., 2016a). Thus, in the context of alpine ski racing, it is not a priori clear which frequencies and signal powers the occurring vibrations possess, and how much of them are actually transmitted to the lower back. Moreover, in alpine ski racing it is so far largely unexplored whether the vibrations acting on the lower back should be considered to be harmless, or whether they might act as potential contributors for developing overuse injuries.

Therefore, the aims of the current study were: (1) to describe power spectral density (i.e., the signal's power distribution over frequency) of the vibrations acting on the different body segments in the competition disciplines giant slalom (GS) and slalom (SL); and (2) to quantify and compare the root-mean-square (RMS) accelerations acting on the lower back (i.e., the severity of vibration exposure) while skiing GS and SL turns.

## Materials and methods

### Measurement protocol and experimental setup

**Figure 1:**
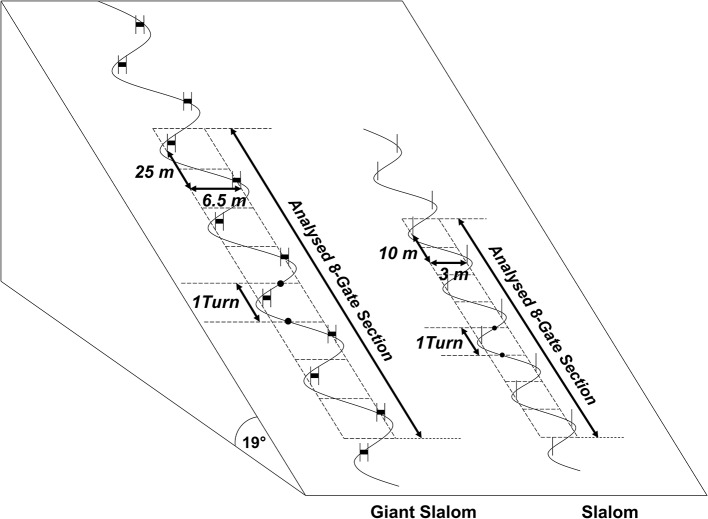
Schematic overview of the experimental on-hill setup.

Six male European Cup-level athletes (85.3 ± 4.9 kg) participated in the study. Within the framework of a biomechanical field experiment, for each athlete the data of two GS runs and two SL runs were collected. For each run performed, an eight-turn section in the middle of a 16 gate-course was considered for further data analysis, resulting in a total 192 included turns (Figure 1). The GS course was set with linear gate distances of 25 m and gate offsets of 6.5 m. The SL course had linear gate distances of 10 m and gate offsets of 3 m. Both courses were set on a constantly inclined slope (19°) with very compact artificial snow conditions, as are typically encountered in the sport of alpine ski racing. Accordingly, on both courses only minor ruts and grooves resulted from the 12 runs performed. The protocol was approved by the ethics committee of the Department of Sport Science and Kinesiology at the University of Salzburg and all subjects gave written informed consent.

### Data collection and instruments

The three-dimensional (3D) accelerations acting on the skier's body segments while skiing were measured at a sampling rate of 500 Hz with six inertial measurement units (Physilog IV; Gait Up; CH) placed on the right and left shanks, right and left thighs, the sacrum and the sternum. The sensors' dimensions were 50 × 39 × 9.2 mm with a 19-gram weight. They were electronically synchronized by radio frequency pulses. In order to minimize the occurrence of any self-resonance and/or soft tissue artifacts, the sensors were fixed to the corresponding body segments on predefined anatomical locations using a skintight custom made underwear suits. For the shank, this was on the medial surface of the tibia bone above the ski boot top and for the thigh, at the mid-distance between the knee and hip joint center (slightly on the lateral side). The sacrum and sternum sensors were fixed directly on the corresponding anatomical landmarks. Additional fixation of the sensors was provided by the athletes wearing their own very close-fitting racing suit. The accelerometers included in the inertial measurement units were set to capture a range of ±16 g and were calibrated following the procedure of Ferraris et al. (1995). To align the sensor frames with the anatomical frames of the body segments, before each analyzed run, a functional calibration procedure consisting of upright still standing, slow squats, vertical trunk rotation and hip abduction and adduction movements was performed. The anatomical frames were defined in accordance to the guidelines of the International Society of Biomechanics (Wu and Cavanagh, 1995). All data processing, parameter computation and statistical analysis steps were performed using the software *MATLAB R2012b* and/or *IBM SPSS Statistics 22*.

### Data processing and parameter computation

During analog-to-digital conversion, all acceleration and angular velocity raw data was low-pass filtered at IMU manufacturer-predefined cut-off frequencies of 94 and 98 Hz, respectively. In order to automatically segment each run and to extract the relevant eight-turn section, 3D segment orientations and a 3D body segment model were calculated as described in detail in previous studies (Fasel et al., 2016b, 2017). For each time instance, the distances between the athlete's center of mass and the left and right ankle joint centers were computed. Turn switches were defined as the crossing points of these two distances, as suggested and validated by Fasel et al. (2016c). Inertial acceleration was computed by transforming the measured acceleration in the global frame, removing the gravity component, and transforming the resulting acceleration back into the anatomical frame.

Power spectral density (PSD) was estimated with the single-sided amplitude spectrum (SSAS) of the inertial acceleration. First, the amplitude spectrum (AS) was computed as the square of the norm of the Fast Fourier Transform (FFT) coefficients of the inertial acceleration along the segment's longitudinal axis. Second, to obtain the SSAS, AS was normalized by the sampling frequency and total number of FFT coefficients and was multiplied by two. For illustration purposes, the final PSD was obtained by smoothing SSAS with a moving average of length 5 and interpolating it between 0.5 and 75 Hz in 0.1 Hz steps.

**Figure 2:**
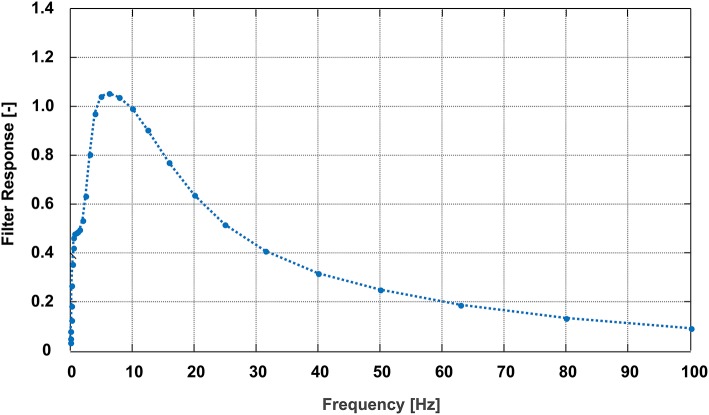
Specifications of the frequency weighted ISO filter used for the calculation of root-mean-square acceleration in accordance with the international standard *ISO 2631* ISO (1997).

Root-mean-square acceleration (RMS) acting on the lower back (i.e., sacrum sensor) during the analyzed eight-turn section was determined based on the inertial acceleration data along the sacrum's longitudinal axis. In accordance with the international standard *ISO 2631* (ISO, 1997), the inertial acceleration data was filtered in the frequency domain prior to computing the RMS according to the ISO filter specifications [frequency weighting Wk (vertical direction) with *k* = 1]. This filter amplifies accelerations at frequencies close to the resonant frequency of the spine [~4–10 Hz according to Izambert et al. (2003), Guo et al. (2009), Guo et al. (2011), and Baig et al. (2014); (Figure 2)]. RMS was then equal to the RMS value of this filtered acceleration.

Following this procedure, for each run and athlete, one PSD curve and one RMS value were obtained. For providing more representative subject/competition discipline curves and values, finally, the PSD curves and RMS values of two eight-turn sections performed by the same athlete and in the same competition discipline were averaged.

### Statistical analysis

The statistical analysis consisted of the following steps: (1) for each body segment and competition discipline, group average PSD curves were computed based on the aforementioned six representative subject average PSD curves; (2) these group average PSD curves were visualized as the areas of uncertainty around the estimate of the mean (i.e., ± the standard error (SE) boundaries); (3) for each competition discipline, group average RMS accelerations acting on the lower back (i.e., sacrum sensor) were calculated based on the aforementioned six representative subject average RMS values and, subsequently, were reported as mean ± standard deviation (*SD*); and (4) potential differences in the lower back (i.e., sacrum sensor) RMS values between GS and SL were tested using a paired sample *t*-test (level of significance: *p* < 0.05), and effect sizes (Cohen d) were calculated.

## Results

**Figure 3:**
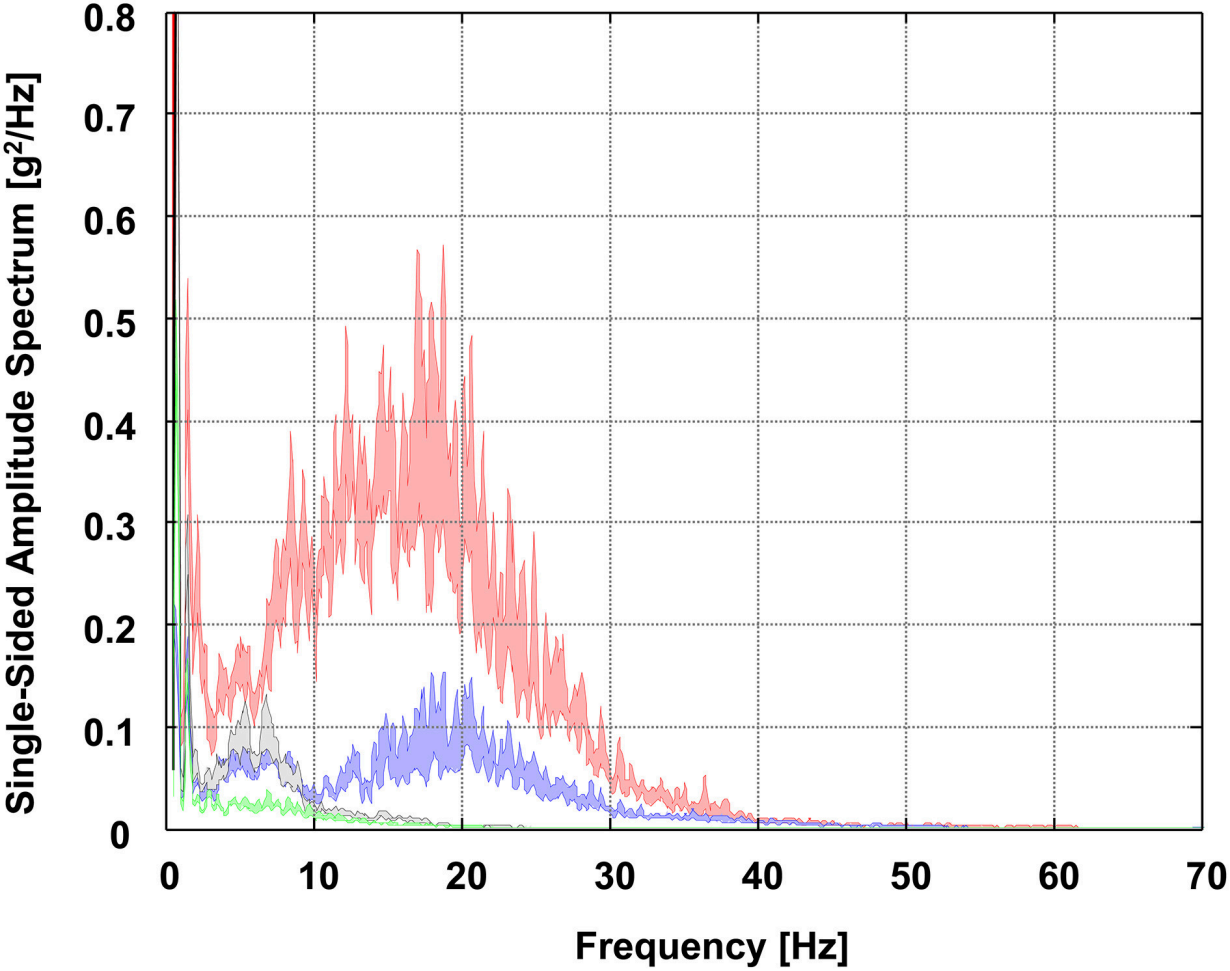
Group average power spectral density (PSD) curves of all segments in GS skiing visualized as the area of uncertainty around the estimate of the mean (±SE). Red, right shank sensor; blue, right thigh sensor; gray, sacrum sensor; green, sternum sensor.

**Figure 4:**
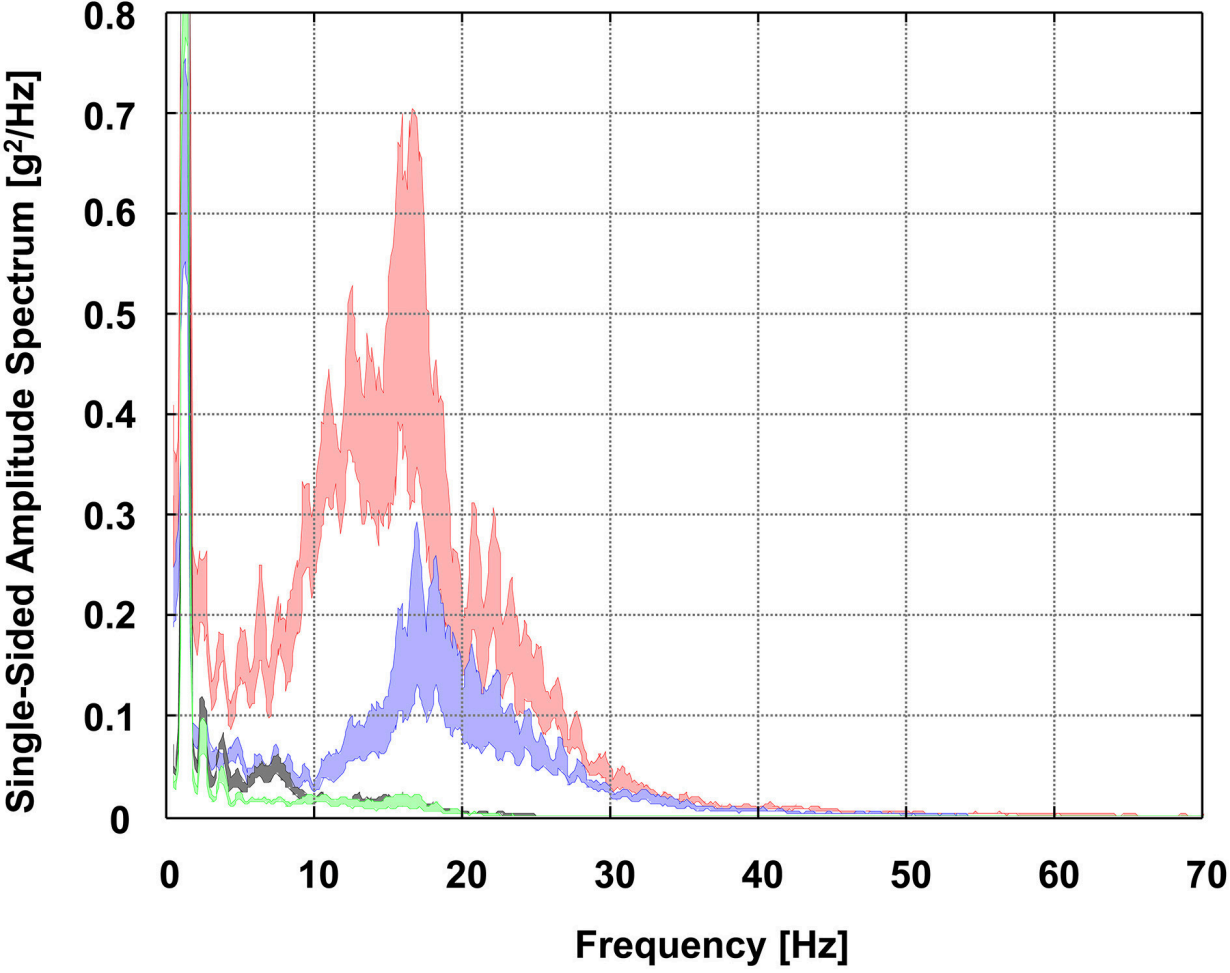
Group average power spectral density (PSD) curves of all segments in SL skiing visualized as the area of uncertainty around the estimate of the mean (±SE). Red, right shank sensor; blue, right thigh sensor; gray, sacrum sensor; green, sternum sensor.

The group average PSD curves of all segments representing GS and SL skiing are depicted in Figures 3, 4. Generally, in both GS and SL, the PSD values of the vibrations acting on the shank were largest for frequencies below 30 Hz. While being transmitted through the body, vibrations were found to be attenuated by each joint (i.e., vibrations at the shank sensor > thigh sensor > sacrum sensor > sternum sensor). Moreover, while at the shank sensor and thigh sensor, PSD values were especially pronounced for frequencies between 10 and 20 Hz; at the lower back (i.e., sacrum sensor), between 4 and 10 Hz PSD values were particularly high. Comparatively, small PSD values were observed at the sternum sensor. At frequencies of below 4 Hz, in the PSD curves of all segments another peak was observed.

**Figure 5:**
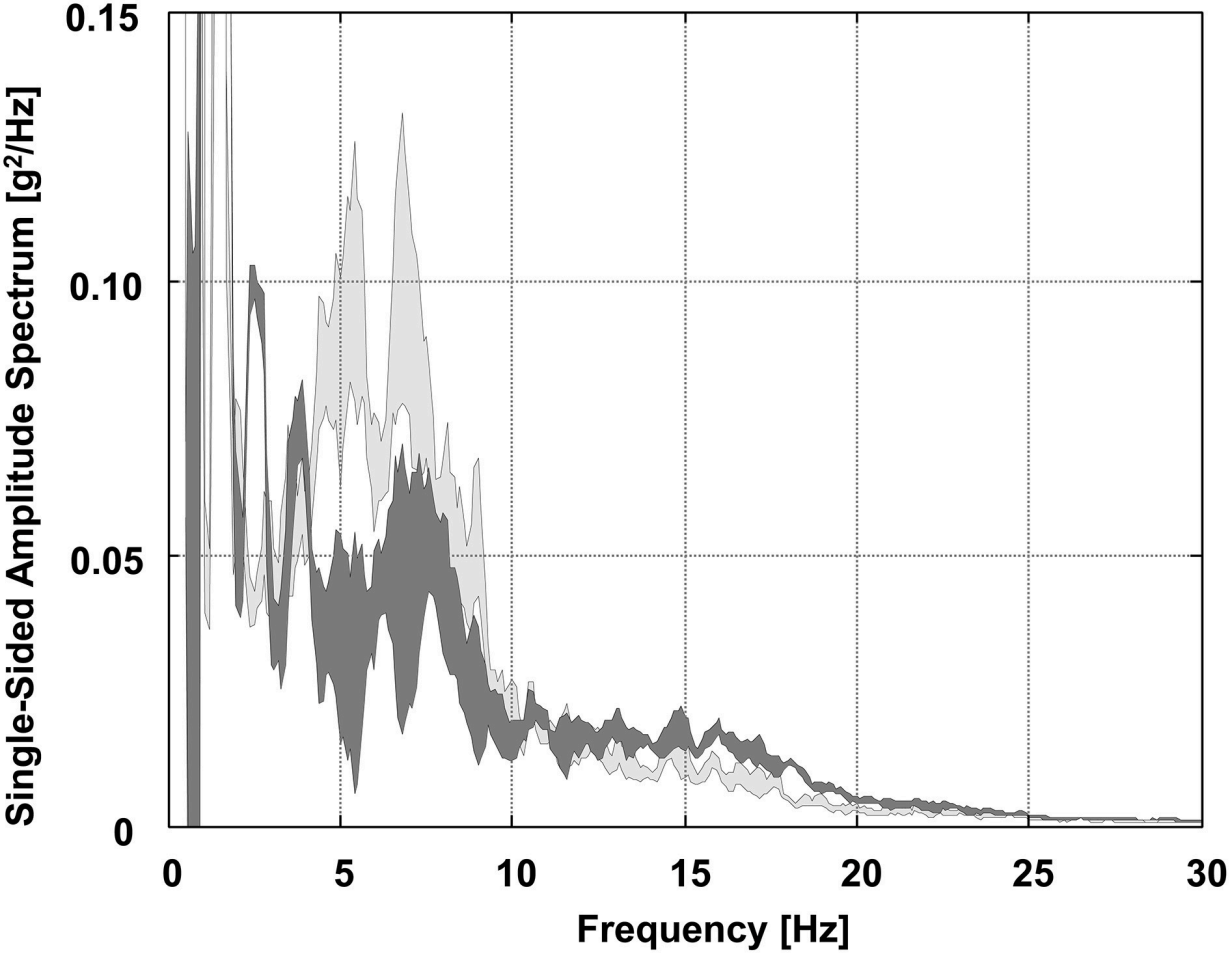
Areas of uncertainty around the estimate of the mean (±SE) of the lower back (i.e., sacrum sensor) group average power spectral density (PSD) curves for frequencies below 30 Hz in GS and SL. Light gray, GS; dark gray, SL.

The PSD curves that explicitly illustrated the vibrations that acted on the lower back (i.e., sacrum sensor) in GS and SL are presented in Figure 5. At frequencies between 4 and 10 Hz, PSD values and, therefore, signal powers of the vibrations acting on the lower back were larger in GS than in SL. Lower back (i.e., sacrum sensor) RMS values were found to be 11.10 ± 1.20 m/s^2^ in GS and 9.35 ± 0.77 m/s^2^ in SL, whereas these values significantly differed at *p* < 0.001 (Table 1).

**Table 1:** Descriptive and inferential statistics of the root-mean-square accelerations (RMS) that act on the lower back (i.e., sacrum sensor) in the competition disciplines giant slalom (GS) and slalom (SL).

**Parameter**	**Mean** ± ***SD***		***t*****-test**	
	**Giant slalom (GS)**	**Slalom (SL)**	***p*-value**	**Cohen d**
RMS [m/s^2^]	11.10 ± 1.20	9.35 ± 0.77	0.001^***^	2.822

Level of significance:

*** *p < 0.001*.

## Discussion

### PSD of the vibrations acting on different body segments in GS and SL

As observed previously for recreational skiing (Federolf et al., 2009; Supej, 2013), in both GS and SL skiing the PSD values of the vibrations acting at the level of the shank sensor were found to be largest for the frequency range below 30 Hz (Figures 3, 4). In this context, it is worth discussing that PSD peaks within this particular range might have different origins. PSD peaks below 4 Hz can most likely be ascribed to the frequency of turns and/or the skier's basic movement patterns. For GS, previous studies revealed turn frequencies of 0.7 Hz and basic movement frequencies of 1.4 Hz, while for SL, turn frequencies of 1.1 Hz and basic movement frequencies of 2.2 Hz were observed (Reid, 2010; Spörri et al., 2012, 2016a). PSD peaks above 4 Hz are most likely a direct consequence of uneven or bumpy snow surfaces and the chattering of the skis when interacting with the snow surface while turning. In this context, it is already known that ski chattering and, therefore, vibrations around 15 Hz to 25 Hz are strongly dependent on the skier's turn technique (skidding vs. carving), the ski's sidecut, and the occurring snow conditions (Kugovnik et al., 2000; Federolf et al., 2009; Supej, 2013).

Starting from the aforementioned vibrations acting on the shank, in both GS and SL vibrations were found to be successively attenuated while being transmitted through the body (Figures 3, 4). While the knee joint mainly attenuated the signal power of all occurring vibrations, the hip joint damped the vibrations, particularly at frequencies >10 Hz, which is in line with previous findings of a pilot study in GS skiing (Fasel et al., 2016a) and fundamental studies under laboratory conditions (Rubin et al., 2003; Kiiski et al., 2008). A distinctive attenuation of ski racing-specific vibrations at frequencies between 4 to 10 Hz, was performed by the spinal structures between the sacrum and sternum sensors. Thus, knowing that vibrations of those frequencies (i.e., close to the resonant frequency of the spine) are the most damaging vibrations for spinal structures and increase the risks of developing low back pain (Hill et al., 2009; Burström et al., 2015), they may be considered potential components of mechanisms leading to overuse injuries of the back in alpine ski racing. Accordingly, special emphasis should be placed on controlling and/or reducing them to a minimum (Griffin, 2004), and protecting athletes by adequate prevention measures. This consideration especially applies to youth athletes whose bodies are still in growth stages.

### Vibration exposure of the lower back while skiing GS and SL turns

Comparing the competition disciplines GS and SL, distinct differences regarding the vibrations acting on the lower back (i.e., sacrum sensor) were identified: for the back overuse-relevant frequencies of 4 to 10 Hz, PSD values were apparently larger in GS than in SL (Figure 5). Moreover, lower back (i.e., sacrum sensor) RMS values, for which calculation accelerations in the range of 4 to 10 Hz are particularly more weighted, were found to be significantly larger for GS than SL (Table 1). This might be explained by the larger average angle between the ski axis and the instant direction of motion (i.e., higher amount of skidding) in GS than in SL (Reid, 2010; Spörri et al., 2012) and, therefore, the more intense vibrations that result when the skis slide more transversally (and less longitudinally) over damaged and/or bumpy snow surfaces. A skidding-induced increase of “usual” chattering of the skis when interacting with undamaged and/or smooth snow surfaces might not serve as an explanation, because this phenomenon is known to be typically related to frequencies around 15 to 25 Hz (Supej, 2013). However, whether the observed competition-discipline specific differences are of clinical relevance needs to be verified by future studies combining both health and load monitoring.

### Methodological considerations

The current study provided valuable insights on the vibrations acting on the human body in GS and SL skiing from a general and a back overuse injury prevention perspective, though there is a potential limitation that needs to be considered when interpreting the study findings. Since the IMU sensors were fixed on the skin and not directly on the bones, particularly for the thigh segment, relative movements between the IMU sensors and the underlying bones might have occurred. These relative movements mainly can be ascribed to soft tissue artifacts, relative displacements of the fixation suit and the resonance of the attached sensors. As a consequence, peak accelerations may be overestimated by ~12%, as it was estimated in a previous study comparing the accelerations measured by skin-fixed and bone-fixed sensors (Kim et al., 1993). However, in view of the major challenges when collecting kinematic data under field conditions and on an alpine ski racing course, a bone fixation was not a feasible option for the current study.

## Perspectives

### Load monitoring in alpine ski racing with body worn sensor technology

One approach for keeping the occurrence of lower back vibration exposure of athletes, and in particular that of youth athletes, within a minimal or healthy dose might be found in the systematic management of training load and recovery time. For that purpose, both continuous load monitoring and a profound injury monitoring are fundamental, implying an evident need for precise assessment tools (Soligard et al., 2016). In the near future, sensor-based wearable technologies might serve as an essential tool, especially for monitoring the cumulative exposure to external loads. In the context of overuse injuries of the back and alpine ski racing, the IMU sensor-based methodology used in this study objectively illustrates the great potential such technologies can have.

On the one hand, with the use of only one IMU sensor, it might be possible to quantify the overall severities of lower back vibration exposures during entire training sessions and/or to specifically monitor vibrations at dangerous frequencies. On the other hand, with the use of two IMU sensors and pressure insoles, it might be feasible to assess the overall trunk movement components and peak loads (enabling a rough estimate of the patterns of spinal disc loading) by long-term measurements during regular training. In the context of alpine ski racing, such an approach has already been applied to short experimental trials under field conditions (Spörri et al., 2015b, 2016a); indicating the small remaining gap toward a direct real-time biofeedback during regular training sessions and or competitions.

### Where to go from here?

Nevertheless, for finding broad application in sport practical settings, there are several preceding steps that need to be taken: from an engineering perspective, body worn sensor technologies still need to be optimized regarding their size, fixation and usability, as well as their real-time and embedded data-processing. In addition, custom-made and application-specific algorithms that take advantage of the characteristics of the specific movement analyzed need to be developed. Finally, prior to the wearable devices/algorithms being launched on the market, rigorous and independent validation and reliability studies are indispensable (Halson et al., 2016; Sperlich and Holmberg, 2016). From a scientific perspective, future research should primarily focus on investigating the relationship between sport-specific external loads and injury risks in order to be able to identify the most relevant parameters for monitoring purposes, and to verify their predictive validity.

In a working-related context, the evaluation of exposures to whole-body vibration is based on the calculation of daily exposure expressed as either: (i) an equivalent continuous RMS acceleration over an 8 h period, or (ii) the vibration dose value (VDV) (Griffin, 2004). Such single measures with corresponding action/limit criteria might serve a more intuitive and perhaps “more coach friendly” approach than the PSD analyses presented in this study. Thus, also in a sports-related context such measures might work. The only missing steps are the definition of sport-related testing protocols and the exploration of appropriate action/limit criteria, which indispensably need to be associated with exposure time. However, as it was nicely illustrated in Griffin (2004), there is a large internal inconsistency within the Directive 2002/44/EC of the European Union for short duration exposures to whole-body vibration, for instance. In this case, the aforementioned two alternative methods (RMS and VDV) may give very different action/limit values. Accordingly, it might appear more prudent to base actions on the qualitative guidance (i.e., reducing risk to a minimum) rather than only refer to the contradicting quantitative guidance values (Griffin, 2004). Catching up this line of argumentation, also in sports-related context, it might be a reasonable alternative approach to just monitor the vibrations acting on the lower back and try (regardless of exposure time) to reduce them to a minimum.

## Conclusion

The findings of this study lead to the conclusion that in addition to the previously suggested combined occurrence of frontal bending, lateral bending and torsion in the highly loaded trunk, the vibrations acting on the lower back also may be considered potential components of mechanisms leading to overuse injuries of the back in alpine ski racing. Accordingly, prevention measures should also aim to control and/or reduce to a minimum the vibrations acting on the lower back while skiing. A particular focus should concentrate on vibrations occurring with a frequency around 4 to 10 Hz because these are known to be the most damaging to the spine. In addition, the current study clearly illustrated the great potential of wearable sensor technologies to monitor and manage the external loads that act on alpine skiers during regular training and/or competitions.

## Author contributions

JS, BF, JK, KA, and EM conceptualized the study design. JS, JK, and BF conducted the data collection. JS and BF contributed to the analysis and interpretation of the data. JS drafted the manuscript, all other authors revised it critically. All authors approved the final version and agreed to be accountable for all aspects of this work.

### Conflict of interest statement

The authors declare that the research was conducted in the absence of any commercial or financial relationships that could be construed as a potential conflict of interest. The reviewer HH declared that he is hosting a Research Topic with one of the authors KA, and the handling Editor states that the process met the standards of a fair and objective review.
